# A Functional MRI Paradigm Suitable for Language and Memory Mapping in Pediatric Temporal Lobe Epilepsy

**DOI:** 10.3389/fneur.2019.01384

**Published:** 2020-01-10

**Authors:** Sarah Buck, Filipa Bastos, Torsten Baldeweg, Faraneh Vargha-Khadem

**Affiliations:** ^1^Cognitive Neuroscience and Neuropsychiatry Section, Developmental Neurosciences Research and Teaching Department, UCL Great Ormond Street Institute of Child Health, London, United Kingdom; ^2^Great Ormond Street Hospital for Children National Health Service Trust, London, United Kingdom; ^3^Unit of Paediatric Neurology and Neurorehabilitation, Woman-Mother-Child Department, Lausanne University Hospital, Lausanne, Switzerland

**Keywords:** fMRI, memory, language, TLE, pediatric, recall, hippocampus

## Abstract

Functional Magnetic Resonance Imaging (fMRI) is a technique frequently used to determine the territories of eloquent tissue that serve critical functions, such as language. This can be particularly useful as part of the pre-surgical assessment for temporal lobe epilepsy (TLE) in order to predict cognitive outcome and guide surgical decision-making. Whereas language fMRI is widely used, memory fMRI is less frequently employed in adult TLE, and lacking in childhood TLE. We have developed a combined language/memory fMRI paradigm that is suitable for children, to provide clinically useful information for surgical planning in pediatric TLE. We evaluated this paradigm in 28 healthy children, aged 8 to 18 years. The advantages of this paradigm are: (a) it examines the functional mapping of language and memory networks within one scanning session, (b) provides assessment of both memory encoding- and retrieval-related neural networks, (c) examines recall-based retrieval to engage hippocampal involvement compared to recognition-based retrieval, and (d) provides overt verbal responses to monitor in-scanner memory performance. This novel fMRI paradigm was designed for language and memory mapping in pediatric TLE and could provide clinically useful information for surgical planning. Finally, parallel versions of the paradigm allow the comparison of brain activations pre- and post-surgical intervention.

## Introduction

Surgical intervention for intractable epilepsy aims to halt or decrease the frequency of seizures (1). However, children with Temporal Lobe Epilepsy (TLE) are at risk of verbal learning and memory deficits after resection of the temporal lobe (2–5). There is a large variability in verbal memory outcome after surgery (6) highlighting the importance of identifying those patients who are at risk of severe memory impairment after temporal lobectomy. Moreover, it has been demonstrated that both short- and long-term verbal memory outcome after surgery in childhood is associated with the integrity of the left temporal lobe (6, 7), suggesting the need for tailored resection of the structures that are critical to memory. Identifying the pattern of language and memory organization prior to surgical intervention could therefore guide tailored resection and limit potential loss of function after surgery.

The self-generated expressive language network is often identified in both healthy children and patients with brain pathologies using a verb generation task, where participants are asked to generate a semantically-appropriate verb for each noun presented. Activated regions typically associated with such a task include Broca's area in the inferior frontal gyrus (IFG), Wernicke's area in the left superior temporal gyrus, the anterior cingulate gyrus, and the dorsolateral prefrontal cortex (8). As with adults, this task typically shows left lateralisation in frontal and temporal regions in children (8, 9). By contrast, the memory network in children has remained relatively unexplored compared to the same network in adults (10–13). Current reports suggest that the memory retrieval network in children is largely similar to that of adults (14, 15), although there is evidence of age-related changes (14). Moreover, despite the identification of task-dependent memory-related brain regions, the hippocampus remains as a central part of the memory network (16–18). Following the documentation of language and memory networks in children, there is a need for functional magnetic resonance imaging (fMRI) paradigms that allow examination of the *interaction* between these two systems. In the face of early brain injury, there is a heightened potential for reorganization, with neural circuits underlying the development of cognitive domains extending to cross circuit interactions to compensate for compromised functions (19).

Early onset seizures interfere with the normal pattern of circuit specialization and hemispheric lateralisation (20, 21). These processes are sacrificed to facilitate neural plasticity and, in turn, to rescue cognitive functions, especially high-priority functions such as speech and language, and verbal memory. Thus, early brain lesions alter the ontogenetic developmental trajectory with the pattern of functional specialization dependent on the age and extent of injury (21).

By virtue of enhanced neural plasticity across development, focal childhood-onset injury results in a pattern of circuit organization that is distinct from that of adult-onset injury. Early onset injury may result in reorganization of memory and language functions to a larger extent than in older patients (6, 22). In patients with TLE who have unilateral lesions, it is difficult to assess how much of their preserved memory is mediated by the unoperated side which can compensate for any failures of the operated side. There is therefore a growing interest in using functional imaging as a pre-operative tool with the aim of identifying the pattern of memory organization, and evaluating the risk of major post-operative memory deficits.

FMRI is a useful pre-surgical tool for language mapping to guide surgical decision making, and predicting cognitive outcome in both adults (23, 24) and children with TLE (25). In pediatric TLE, atypical language lateralisation is relatively frequent (26). It is possible that reorganization of memory function may also co-occur in such cases, as documented in adult TLE, and should be investigated alongside language lateralisation. Whereas memory fMRI is used in adult epilepsy studies (27–30), pediatric studies have not yet investigated memory organization, and have instead focused on identifying language lateralisation as a proxy for memory lateralisation.

Thus, information obtained from language fMRI is sometimes used to predict memory outcome in TLE, due to mesial temporal lobe (MTL) activation during language tasks (31). However, using language fMRI to predict memory outcome assumes co-lateralisation of these functions. Co-lateralisation of language and memory has previously been studied (32), but dissociating these domains of function can be difficult, partly due to reorganization, and to overlapping and/or interconnectivity of regions involved during cognitive processing. Moreover, Sepeta and colleagues demonstrated that whereas healthy adults show co-lateralisation of activation in Broca's area and the MTL during a language task, children do not demonstrate this pattern (31). This suggests that language fMRI may not be a viable substitute to predict memory outcome. There is therefore a need for developing suitable memory fMRI paradigms, as opposed to relying on language fMRI, for the prediction of memory outcome, particularly in pediatric patients. In addition, it is important to examine the relationship between language and memory lateralisation.

In adult studies, memory fMRI paradigms usually involve recognition- rather than recall-based responses (30, 33–35). Lesion studies have provided evidence of the distinction between recall and recognition processes. Patients with developmental amnesia (DA) who sustained selective early-onset bilateral hippocampal pathology (36) exhibit severe and selective impairment in recall memory, in the context of relatively well-preserved recognition memory (37–39). This suggests that fMRI paradigms that use recognition-based responses are more likely to be insensitive to recall-based (i.e., hippocampal) activation. Moreover, adult studies employing multiple levels of deep vs. shallow processes, such as the recognition tasks based on Remember/Know decisions, may be too complex for children. Given that children with TLE demonstrate difficulty in learning and recall of new information (35, 40), it is more informative to use a recall- rather than a recognition-based memory paradigm.

In an effort to meet the needs of the fMRI community, this study presents a novel fMRI paradigm for the functional mapping of language and memory, *within one scanning session*, to guide surgical decision-making and help with predictions of outcome. The paradigm was developed with the following goals:

- Design a paradigm sensitive to MTL function because of its known involvement in episodic memory and its susceptibility to pathology in TLE.
- Provide a combined language/verbal memory fMRI paradigm to examine the interaction of the two networks within one scanning session, thereby facilitating a cost- and time-effective investigation.
- Examine hippocampal activity related to both memory encoding and retrieval.

Several variables related to the experimental fMRI paradigm will be specifically outlined in the results section to test paradigm validity and reproducibility.

## Materials and Methods

### Participants

Thirty normally-developing, English-speaking children and adolescents were recruited through East London schools. Using the standard exclusion criteria (movement that exceeds 3 mm or 2°), two participants (one male and one female) were excluded from further analyses due to high level of in-scanner movement (Supplementary Figure S1). The sample includes 11 males and 17 females, aged between 8 and 18 years (*M* = 14, *SD* = 3). Handedness was measured for each participant using the Edinburgh Handedness Inventory (41). The scores were representative of the sampling population: two participants were left-handed, one was ambidextrous. Socio-economic status (SES) was determined for each participant with deprivation deciles ranging from most deprived (score of 1) to least deprived (score of 10). SES deciles in the present cohort ranged from 2 to 10 (*M* = 5, *SD* = 2). Table 1 illustrates the participants' demographics. Written informed consent was obtained from each participant prior to study start.

**Table 1:** Participants' demographics (*N* = 28).

	**Mean**	**Min**	**Max**
Age in years (M ± SD)	14 (3.0)	8	18
Gender (M/F)	11/17	N/A	N/A
Atypical handedness	3 (11%)	N/A	N/A
SES (M ± SD)	5 (2.0)	2	10
Full scale IQ (M ± SD)	108 (8)	90	126

### Neuropsychological Assessment

Intellectual status was assessed using the Wechsler Abbreviated Scale of Intelligence—Fourth Edition (WASI-IV). This test provides measures of full scale IQ (*M* = 108, *SD* = 8), verbal IQ (*M* = 108, *SD* = 8), and performance IQ (*M* = 107, *SD* = 10).

Verbal learning was assessed using the Word-Pair subtest of the Children's Memory Scale (CMS). This is a widely-used standardized diagnostic tool for memory in children. The Word-Pairs subtest of the CMS assesses the ability to learn a list of pairs of words over three consecutive trials, whereby the examinee is presented with the first word of each pair and is asked to recall the second word (cued recall). Following a 30 min delay, the participant is asked to retrieve as many word-pairs as possible, first through free recall, then through cued recall by presenting the first word of the pair, and finally through yes/no recognition judgments of each word pair to indicate whether they were part of the list that was learned earlier. Learning and memory scores are presented in Supplementary Table S2.

### The Novel fMRI Paradigm

According to the levels of processing effect, deep processing of information (e.g., encoding the meaning of an item) leads to better subsequent retrieval than shallow processing (e.g., encoding the perceptual features of an item) (42). As such, a verb generation task, which involves generating a verb related to a noun heard, may be used as a deep encoding task. This paradigm comprises a noun-to-verb generation task for deep encoding (i.e., memory encoding), and a subsequent recall task of the nouns (i.e., memory retrieval). Therefore, this paradigm combines language and memory mapping within one scanning session.

#### Language Task: Verb Generation

Verb generation tasks produce strong and consistent lateralised activation in the left hemisphere language network and are the standard tasks used in the clinic (8, 43). During the verb generation task used here, participants were presented with nouns, one at a time, and were asked to overtly generate a verb for each noun (for example they heard “cake” and generated the verb “eating”). There were a total of 60 nouns, divided into 6 lists of 10 each.

#### Memory Task: Cued Recall

The memory task required the participants to overtly recall the nouns that were presented during the language task. Two-phoneme word stem cues were presented one at a time to the participants to guide recall of previously encoded words (for example “æn” as a cue for “animal”). Participants were asked to say the word it corresponded to, or say “pass” if they could not retrieve the word. Each stem was unique in the full list of study words (44).

Cued-recall using word-stems has multiple advantages. First, it allows event-related investigation of fMRI data, as retrieval-related activation is time-locked to each cue. This permits examination of brain activation specifically related to memory retrieval success (correctly recalled vs. forgotten). Second, the performance reflects declarative recall which is known to be dependent on the hippocampus (39). This approach has been successfully adopted in previous studies that reported activation in the hippocampus during successful recall (45, 46).

More details about the procedure (e.g., duration and timings) of the language and memory tasks is provided in the procedure section.

##### Accounting for priming effects

Priming is the facilitation in the processing and/or re-evoking of a stimulus due to a prior encounter with that stimulus, and is devoid of intentional and conscious recollection (47). In word- stem completion tasks, stems are more likely completed with previously presented words. In order to reduce the priming effect in our cued recall task, several control measures were adopted. First, stems for words that were not previously heard (foils) were inserted in the cued recall phase (15 words in each list: 5 foils and 10 target words), to which participants were expected to respond by saying “pass.” The stems of these foils did not match any studied words. With this method, it is possible to have a measure of false alarms (i.e., stem completion with non-studied words). Second, each of these unique 90 stems (from 60 studied words and 30 foils) was shared with at least 4 other common words, thus requiring conscious recollection to retrieve the correct word. Using these methodological considerations, the risk of priming effects was minimized, and performance was expected to primarily reflect conscious recall.

Foils that were erroneously completed with a word, instead of a “pass” response, were categorized as “false alarms.” Performance was calculated as percent correct recall, minus false alarms.

#### Baseline Task

The baseline task required making an odd/even decision to numbers; for example, the participant was presented with the number “3” and had to say “odd.” The presentation rate of this number was similar to the rate of word and word-stem presentation (*viz*, every 4 s).

This baseline task was designed to meet three goals: First, it acted as a baseline to subtract from the active conditions (Language and Memory) and enable investigation of activation contrasts. The second purpose was to introduce a short delay between encoding and recall (50 s), and the third goal was to prevent subvocal rehearsal and maintenance of information in the short-term memory store during the delay. The selection of this baseline task therefore optimized investigation of brain activation during the language and memory tasks.

#### Stimulus Material

Stimuli were selected from the MRC Psycholinguistic Database. The stimuli matched the ones used for the clinical verb generation paradigm, currently the protocols of choice at Great Ormond Street Hospital, according to several features: word frequency (48), concreteness, familiarity, and imageability (Supplementary Table S1). In addition, all of the words were simple enough to have been acquired before the age of 8 (49) and were composed of 1 to 3 syllables, similar to the version used in the clinical setting.

#### Overt Response

The present paradigm required overt verbal responses (50) in order to monitor in-scanner performance and to conduct event-related analysis. Moreover, overt speech responses have the potential to reveal the interaction of memory and language networks as the memory item retrieved is translated into a verbal output.

#### Procedure

**Figure 1:**
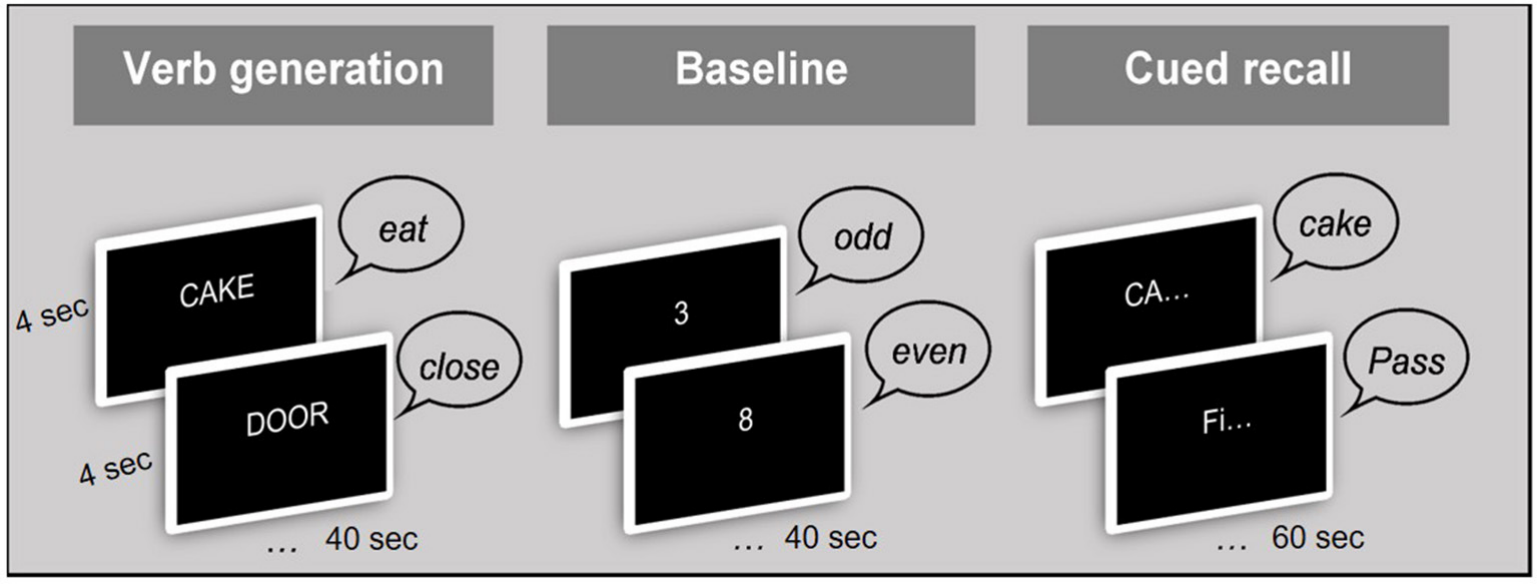
Procedure of the fMRI paradigm.

The scanning session consisted of 3 runs, each with two word-lists. Verbal responses were monitored via an MRI-compatible microphone. Figure 1 illustrates the procedure of the fMRI paradigm. Before the beginning of each block, a visual prompt was displayed on the screen for 5,000 ms in order to prepare the participants for the upcoming task. These prompts were [ACTION WORDS] for the verb generation [ODD OR EVEN?], for the baseline block, and [REMEMBER OR “PASS”] for the cued recall block (Figure 1). The stimuli were presented at a rate of one every 4 s, which was purposely not locked to the TR (1.25 s) in order to improve effective sampling of the signal (51). Each block of verb generation and baseline lasted for 40 s (10 × 4 s), while the cued recall block lasted for 60 s (15 ×4 s), and the entire protocol lasted for 16 min. The presentation of stimuli followed the same order for each participant.

The standardized test of memory function, Children's Memory Scale (CMS) was administered outside the scanner, with a time delay of at least 1 h between the behavioral and the imaging sessions. The duration of the behavioral session (neuropsychological assessments) and the MRI session was approximately 1 h each, and occurred on the same day. The order of sessions depended upon scanner availability.

A subset of the sample (*N* = 15) were administered the same fMRI protocol, 1–2 years after the first session (mean elapsed time between sessions = 1.5 years, *SD* = 0.6), using another version of the paradigm (see Two Parallel Versions). This procedure allows the investigation of test-retest effects.

#### Two Parallel Versions

Two versions of this paradigm were developed using different words from the same database and with the same criteria (see Stimulus Material). The parallel versions allow administration to the same participants at two time points (e.g., before and after surgery). Participants in the current cohort were allocated randomly to one of the two versions (version A, *N* = 13; version B, *N* = 15). In-scanner memory performance is illustrated per fMRI version and per run in Supplementary Table S3.

### Data Acquisition

Data were acquired on a 3T Siemens MRI system with a 20 channel head coil. Imaging parameters for multiband EPI images were the following: TR (repetition time) = 1,250 ms, TE (echo time) = 26 ms, slice thickness 2 mm, slice gap 1 mm. The 40 slices per volume were acquired with interleave. A slice tilt was applied to align the scans perpendicular to the long axis of the hippocampus and optimize the Blood Oxygenated Level Dependent (BOLD) sensitivity in medial temporal lobe regions (52). For each functional scanning run, 270 images were acquired, with a total of 810 images across the 3 runs. In addition to the functional images, a T1-weighted magnetization prepared rapid gradient-echo (MPRAGE) scan was acquired for anatomical localization, with a slice thickness of 1 mm, repetition time of 2,300 ms and echo time of 2.74 ms.

### Data Pre-processing

Spatial realignment of the images was applied using Statistical Parametric Mapping software (SPM12, Wellcome Department of Cognitive Neurology, London, UK: www.fil.ion.ucl.ac.uk/spm/). The images were then unwarped to reduce spatial distortion using the TOPUP toolbox in FSL (53). Additional retrospective motion correction was applied using Functional Image Artifact Correction Heuristic (FIACH) (54). Finally, the images were co-registered, normalized to a standard MNI space for group analyses, and smoothed with a Gaussian kernel of 6 mm full width half maximum (52). We used the adult MNI template from SPM, due to the age variability in our cohort, and the inclusion of older children.

### Image Analyses

Image analyses were conducted using SPM12. Movement parameters were included in the design matrix as covariates. For individual-subject analyses (1st level), the changes in BOLD signal over time were examined for each individual using fixed effect analysis across the three runs. For group analyses (2nd level), contrast estimates from each individual were entered into a GLM with individuals treated as a random factor. Extent and height thresholds were employed, and are specified where appropriate.

#### Statistical Thresholds and ROI Analysis

For analyses without *a priori* hypotheses, whole-brain analysis at the group level is reported, corrected for multiple comparisons (*p* < 0.05 Family-Wise Error (FWE) corrected). For analysis of memory with prior anatomical hypotheses, analyses are reported at threshold *p* < 0.001, uncorrected, in keeping with a previous fMRI study of memory (55). We reduced the number of statistical tests by using a method that exploits anatomical information in the form, or region of interest (ROI) masks. In such masked analysis, only voxels within the mask are included in the analysis. The anatomical constraint in the block, and event-related analyses described below involved a gray matter ROI mask, reducing the number of voxels from 14,000 to 10,000. Moreover, as a result of the known involvement of the hippocampus in delayed-recall memory, this region was of *a priori* interest. As such, hippocampal activations were corrected for multiple comparisons, using a small volume correction (56) within the hippocampus ROI (*p* < 0.05, FWE corrected). To illustrate hippocampal activation after small volume correction, group analyses were repeated within a hippocampal mask (*p* < 0.05 FWE corrected) and are displayed in Supplementary Figure S4.

#### Block Analysis

Three regressors of interest were created: Language, Baseline and Memory (Table 2). Language activations were investigated for the contrast “Language vs. Baseline.” Whole-brain analysis at the group level is reported at a height threshold of *p* < 0.05, corrected for multiple comparisons (FWE correction).

**Table 2:** Description of each regressor of interest.

	**Regressors**	**Description**
Block analysis	Language	Verb generation task
	Baseline	Baseline task: odd/even decision to numbers
	Memory	Cued recall task, irrespective of performance
Event-related analysis	Subsequent Hits	Activation during the encoding of words that were later retrieved
	Subsequent Misses	Activation during the encoding of words that were later forgotten
	Baseline	Baseline task: odd/even decision to numbers
	Hits	Activation during the successful retrieval of words
	Misses	Activation during the unsuccessful retrieval of words
	Correct rejection	Activation during correct rejections of words at retrieval

Memory encoding activations were investigated for the contrast “Language vs. Baseline.” Memory retrieval activations were investigated for the contrast “Memory vs. Baseline.” Whole-brain analysis at the group level is reported at a height threshold of *p* < 0.001, uncorrected. Small volume corrections (*p* < 0.05 FWE corrected) within the hippocampus were subsequently applied (see section Statistical Thresholds and ROI Analysis).

#### Event-Related Analysis

Six regressors of interest were created (Table 1): Subsequent Hit, Subsequent Misses, Baseline, Hits, Misses, and Correct Rejections. *Memory encoding success* (also known as the subsequent memory effect) was examined by comparing activation for words that were subsequently remembered (Subsequent Hits) to activation for words that were subsequently forgotten (Subsequent Misses) (contrast Subsequent Hits vs. Subsequent Misses). *Memory retrieval success* was examined by comparing activation for words that were remembered (Hits) to activation for words that were forgotten (Misses) and for words that were correctly identified as “new” (Correct Rejections) (contrast Hits vs. Misses & Correct Rejections).

Whole-brain analysis at the group level is reported at a height threshold of *p* < 0.001, uncorrected. Small volume correction (*p* < 0.05 FWE corrected) within the hippocampus were subsequently applied (see section Statistical Thresholds and ROI Analysis).

#### Laterality Indices

Lateralisation indices (LI) assess hemispheric lateralisation for a specific cognitive function. This LI was calculated based on the sum of voxel values in each hemisphere (57). Consistent with clinical studies, values above 0.2 are considered left lateralised, LIs below −0.2 are considered right lateralised, and values between −0.2 and 0.2 indicate bilateral representation.

For the present purpose, LIs were calculated in two ROIs; in Broca's area and in the hippocampus. Language lateralisation was determined based on LI values in Broca's area during the verb generation task, and memory lateralisation was determined in the hippocampus, based on group-level analysis that generated the strongest hippocampal activation (see Group-Level Activations), that is, memory encoding with block analysis and memory retrieval with event-related analysis. The distribution of language and memory LIs is illustrated in Supplementary Figures S2C–E.

### Test Validity

#### Memory Performance Between the Two fMRI Versions

In-scanner memory performance was compared between the two fMRI versions, using an independent sample *t*-test, to examine (a) the feasibility of combining the two versions for subsequent analyses, and (b) the utility of these tools for comparable assessment across two time points.

#### In- and Out-of-Scanner Memory Performance

Performance on the task administered inside the scanner was compared to performance on a standardized test of memory administered outside the scanner, i.e., learning and delayed recall of Word-Pairs from the CMS. For the purpose of this correlation analysis, raw scores in percentages from the CMS, rather than the standardized scores, were used for better comparison with in-scanner memory performance.

#### Effect of In-scanner Movement on Data Quality

The impact of movement parameters (from the FIACH toolbox) on EPI mean image intensity was investigated. In-scanner motion can degrade image quality and reduce signal-to-noise ratio (SNR) (58). The effect of movement artifacts was therefore investigated in the hippocampus ROI due to its susceptibility to low SNR. Correlations were computed between signal intensity and FIACH temporal SNR (tSNR), which is a measure of deviation of the realigned images (54). The EPI mean signal intensity in the hippocampus was normally distributed (Shapiro-Wilk *p* = 0.182), and so was FIACH temporal SNR (Shapiro-Wilk *p* = 0.184); therefore, we proceeded with a Pearson correlation. Distribution of hippocampal signal intensity and FIACH tSNR are illustrated in Supplementary Figures S2F,G, respectively.

#### Age Effect on In-scanner Behavioral Performance

Due to the large age variability in the current sample, we tested the effect of age on in-scanner language scores (controlling for non-verbal IQ) and memory scores (controlling for full scale IQ), using partial Pearson Correlations. This verifies the usage of the fMRI paradigm across the age range of our sample. Distribution of in-scanner language and memory scores is illustrated in Supplementary Figures S2A,B, respectively.

#### Age Effect on Functional Lateralisation

We tested the effects of age on functional lateralisation for language and memory, using Pearson Correlations.

### Reproducibility of the Paradigm

#### Memory Performance Across Runs

The reproducibility of the paradigm was determined based on the stability of the behavioral data across the three scanning runs, which were acquired a few minutes apart. For this section, each run was analyzed separately to investigate inter-run variability.

The consistency between performance across runs was measured using Intra Class Correlation (ICC), which is a measure of the ratio of between-subject variance and between-tests variance. In this respect, the value approaches 1 if the variability across individuals is larger than the variability within individuals across repeated runs. The ICC was based on a 2-way mixed-effects model.

#### Signal Intensity in the Hippocampus Across Runs

Signal intensity in the hippocampus was identified in each individual's EPI mean acquisition and compared across scanning runs. Signal intensity in a control region, the cingulate cortex, was also compared across scanning runs.

#### Laterality Indices (LIs) Across Runs

The consistency between LI values across runs was measured using ICC, based on a 2-way mixed-effects model.

#### Laterality Indices (LIs) Across Sessions

The consistency between LI values across two separate sessions (time 1 and time 2) was measured using ICC, based on a 2-way mixed-effects model.

## Results

### Group-Level Activations

#### Language Activations

**Figure 2:**
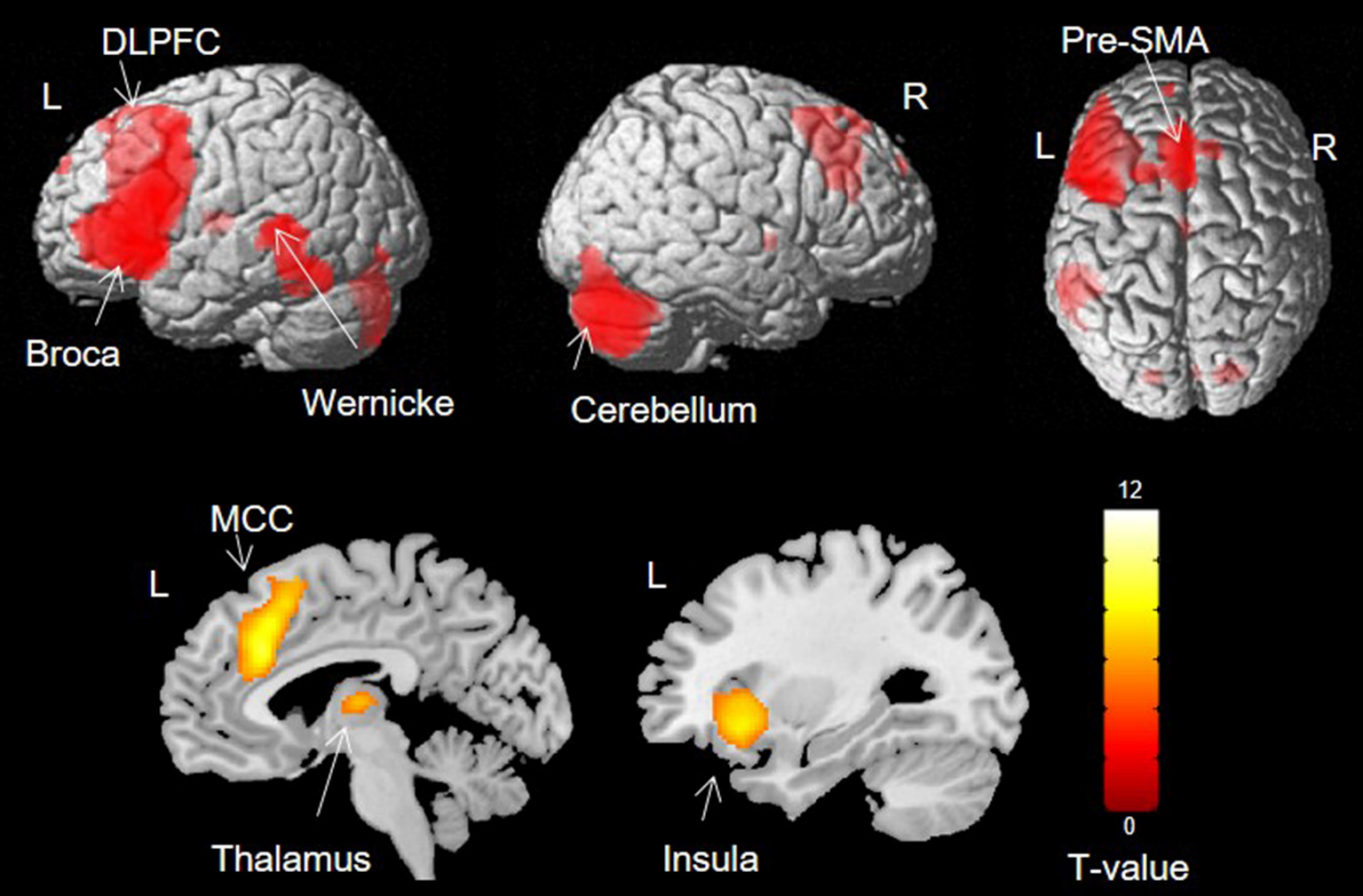
Group activation during verb generation task (*p* < 0.05, FWE).

Activation was found in left Broca's area, the left STG, bilateral dorsolateral prefrontal cortex (DLPFC), pre-supplementary motor area (pre-SMA), right cerebellum, left thalamus, left anterior insula and bilateral middle cingulate cortex (MCC) (Figure 2).

#### Memory Activations

**Figure 3:**
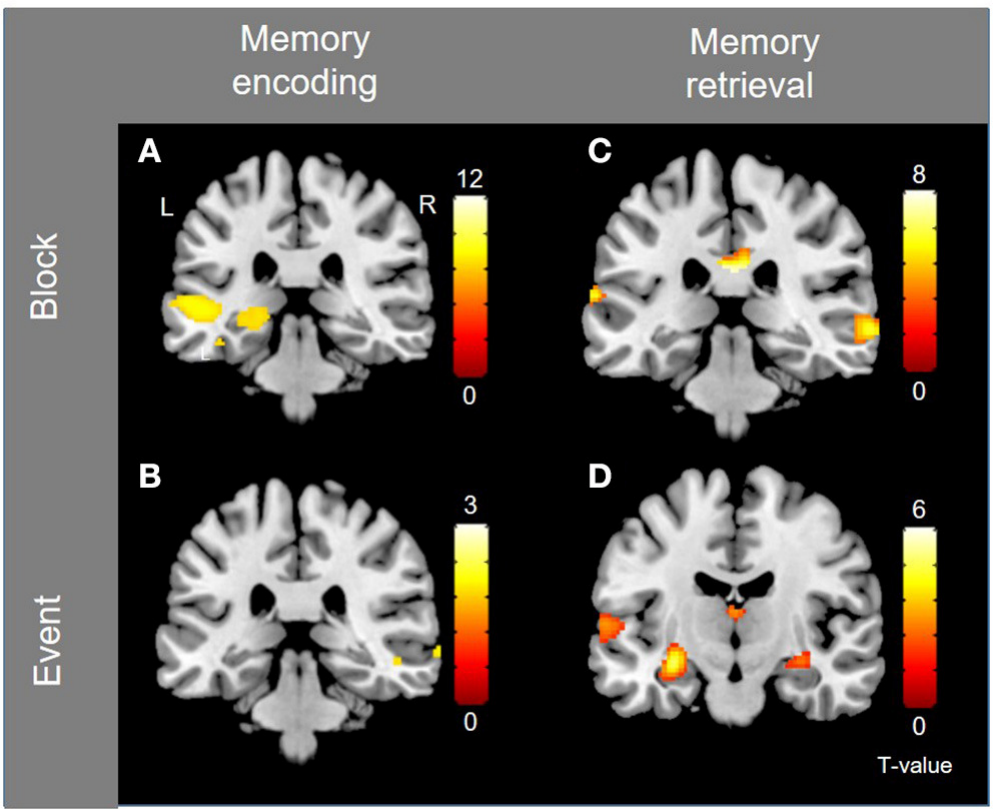
Group-level activations for memory encoding and retrieval, for block- and event-related analyses, separately. **(A)** Block-activation for subsequent memory (i.e., memory encoding). **(B)** Event-related activation for subsequent memory (i.e., memory encoding). **(C)** Block-activation for memory retrieval (contrast Memory vs. Language). **(D)** Event-related activation for successful retrieval. Activations are shown with threshold of *p* < 0.001, uncorrected.

Block-level whole-brain activation associated with memory encoding (contrast Language vs. Baseline) is documented in the previous section (i.e., language activations), and is illustrated in Figure 3A, where left hippocampal activation is observed. The small volume correction resulted in significant activation within the left hippocampus in three separate peaks (1: peak coordinates −28 −28 −6, T = 4.30, corrected *p* = 0.011, 2: peak coordinates −20 −30 −4, T = 3.95, corrected *p* = 0.030, and 3: peak coordinates −14 −36 2, T = 3.82, corrected *p* = 0.043). Event-related activation associated with memory encoding success (contrast Subsequent Hits vs. Subsequent Misses) was shown in the left temporal pole and right posterior superior temporal lobe, shown in Figure 3B.

Block-level activations for memory retrieval (contrast Memory vs. Baseline) were found in left Broca's area, left dorsolateral PFC, bilateral cerebellum and bilateral posterior temporal lobes. Activations were also shown in bilateral anterior insula, bilateral pre-SMA, bilateral middle and posterior cingulate cortex (PCC & MCC), and bilateral caudate nuclei. Because many of these activations overlap with those reported for language, another contrast was investigated (contrast Memory vs. Language) to identify activations that are specific to the memory task. Activations were shown in right dorsolateral PFC, right orbitofrontal PFC, bilateral posterior temporal lobes (right posterior middle temporal gyrus and left posterior STG), right pre-SMA, and posterior cingulate cortex (*p* < 0.001, uncorrected), as shown in Figure 3C. Event-related activity associated with memory retrieval success (contrast Hits vs. Misses & Correct Rejections) was shown in bilateral hippocampi, left posterior STG and left caudate (Figure 3D). The small volume correction resulted in significant activation within the left hippocampus (peak coordinates −30 −14 −12, T = 4.38, corrected *p* = 0.005).

Left hippocampal activation is shown during encoding (block analysis) and retrieval (event-related analysis) after small volume correction and is illustrated in Supplementary Figure S4. Group-level analyses were repeated with age and gender as nuisance regressors, and yielded similar results (Supplementary Figure S5).

### Test Validity

#### Memory Performance Between the Two fMRI Versions

Memory performance was not significantly different between versions A (54%) and B (60%) [*F*_(1, 26)_ = 0.198, *p* = 0.504]. Memory scores from the two versions were therefore collapsed for the subsequent analyses.

#### In- and Out-of-Scanner Memory Performance

For the purpose of this analysis, in-scanner memory performance was collapsed across fMRI versions (see Memory Performance Between the Two fMRI Versions) and runs (see Cued-Recall Performance Across Runs). Cued recall scores on the fMRI task were moderately correlated with CMS learning scores (*r* = 0.45, *p* = 0.019), but not with CMS delayed recall scores (*r* = −0.025, *p* = 0.903).

#### Effect of In-scanner Movement on Data Quality

No significant relation was found between in-scanner movement parameters and signal intensity in the hippocampus (*r* = −0.104, *p* = 0.613).

#### Age Effect on In-scanner Behavioral Performance

Partial correlations showed that age was significantly correlated with language scores (*r* = 0.39, *p* = 0.048), and memory scores (*r* = 0.47, *p* = 0.019) where older children performed better than younger children. However, the correlation between age and language scores was moderate and no floor/ceiling effect were shown in any of the measures.

#### Age-Effects on Functional Lateralisation

Age was not significantly related to functional lateralisation of language (*r* = −0.18 *p* = 0.380), memory encoding (*r* = −0.18 *p* = 0.372) or memory retrieval (*r* = −0.04 *p* = 0.854).

### Reproducibility of the Paradigm

#### Cued-Recall Performance Across Runs

Performance accuracy was 56% (*SD* = 35) in the first run, 53% (*SD* = 26) in the second run, and 58% (*SD* = 21) in the third run (Supplementary Figure S3A). ICC was 0.92, indicating good stability of performance across runs. This implies that it is possible to collapse findings from across the runs and treat them as fixed effect in 1st level analyses.

#### EPI Mean Signal Intensity in the Hippocampus Across Runs

Signal intensity in the hippocampus was compared across scanning runs (Supplementary Figure S3B) and was found to be stable. ICC was 0.98, indicating high reliability of mean signal intensity in the hippocampus across runs. ICC for the signal intensity in the cingulate cortex was 0.99, indicating high reliability of mean intensity in the control region as well.

#### fMRI Laterality Indices (LIs) Across Runs

Group-level language and memory LIs were examined across the three runs (Supplementary Figure S3C). For language LIs, ICC was 0.44, indicating moderate stability of values across runs. For memory encoding, ICC was 0.37, indicating moderate stability of values across runs, however for memory retrieval ICC was −0.28 indicating low interclass correlation.

#### fMRI Laterality Indices (LIs) Across Sessions

Group-level language and memory LIs were examined across the two sessions in the subset of participants who were scanned twice. For language LIs, ICC was 0.71, indicating good stability of values across sessions. For memory encoding LIs, ICC was −0.71 indicating low interclass correlation. Finally, for memory retrieval LIs, ICC was 0.45, indicating moderate stability of values across sessions.

## Discussion

We designed a novel fMRI paradigm for the mapping of language and memory, and the examination of the interaction between those two systems, in children. Here we discuss the validity of this paradigm based on the performance of a group of healthy children and adolescents. Group-level activations were found in regions typically associated with expressive language. For memory, hippocampal activation was detectable during both memory encoding and retrieval, using block, and event-related analyses. The average memory performance across the runs (56% correct) provides enough trials in each condition (i.e., Hits and Misses) allowing event-related analyses. Validity of the paradigm was demonstrated through moderate correlations between in-, and out-of-scanner memory scores, suggesting that the novel fMRI paradigm relates to memory performance outside the scanner, but is also influenced by other factors inside the scanner, such as testing environment and nature of the test. In-scanner memory scores were correlated with out-of-scanner learning scores, but not with delayed recall scores possibly due to the longer delay interval (i.e., 20 min, as opposed to 50 s in the scanner). Importantly, in-scanner motion, often attributed to overt responses, did not significantly impede fMRI data quality. Notwithstanding a significant age effect on in-scanner language and memory scores, attributed to the trajectory of normal cognitive development, the correlations were moderate and children across the age range studied were capable of performing the tasks.

Reproducibility of the paradigm was tested by examining the stability of cued recall performance and EPI signal intensity in the hippocampus across three scanning runs, as well as the stability of fMRI LIs across three scanning runs and two scanning sessions separated by an average of 1.5 years. These variables showed intra-session stability of language and memory encoding, as well as inter-session stability of language and memory retrieval, providing evidence for the paradigm's reliability and reproducibility.

### Effect of In-scanner Movement on Data Quality

Task-related motion, such as head movement related to speech, can cause signal changes which may hamper data quality and be misinterpreted as brain activation (59). Negative effects of in-scanner motion are especially pronounced in pediatric populations (60) and should be taken into consideration in fMRI studies involving overt speech. Image quality is specifically compromised in images with low SNR (61). Therefore, in the present study, the effect of movement artifact on image quality was specifically investigated in the hippocampus, but found not to have a significant impact on fMRI data quality. This provides evidence that overt speech should be considered in future fMRI studies. FIACH is also very effective in reducing between- and within-volume motion-related effects (54).

### Effect of Age on Functional LIs

In typically-developing children, language lateralisation is emergent by the age of 5 (44, 62), but changes with increasing age parallel the development of language skills (63). However, the present findings do not suggest a developmental trajectory in language and memory lateralisation. It is possible that tracking changes in degree of lateralisation as a function of age requires large cohorts separated by age bands to mirror the stages of cognitive development as compared to one group spanning a wide age range (8 to 18). Similar to language, it is possible that verbal memory lateralises early in life, and administering the memory paradigm in younger children (between the ages of 5 to 8) could potentially shed light on the developmental trajectory of memory lateralisation. Indeed, increasing left lateralisation for language in the MTL across childhood has been shown in a cohort of young children (6–13 years old) (31). To explore this further, age-related changes in memory-related MTL lateralisation should be further explored in children below the age of 8 years, particularly as different aspects of cognitive memory emerge at different stages of development.

### Reproducibility and Reliability

The reproducibility of the fMRI paradigm was tested by investigating the stability of memory performance and EPI mean signal intensity in the hippocampus across the three scanning runs, as well as the stability of language and memory LIs over (a) three scanning runs and (b) two separate sessions. The consistency of performance across the runs indicates that the memory paradigm yields reproducible results.

Intra- and inter-session reliability of LIs was examined by measuring stability of language and memory LIs across three scanning runs, and across two scanning sessions (1.5 years apart on average), respectively. Bennett and Miller suggested a range of ICC values between 0.33 and 0.66 within which fMRI studies are typically reliable (64). As per this range, LI values (for both language and memory) were more stable across scanning sessions than across runs, possibly due to higher statistical power as a result of additional trials (20 trials per run vs. 60 for the whole session). This indicates the importance of having a considerable number of trials to provide a measurable and reliable response.

In the present study, language LIs were stable across runs and sessions, thereby reflecting reliable results. Memory encoding LIs were also stable across runs, but not across scanning sessions. This may be a result of noise in the data (e.g., physiological noise from the participants, and system noise in the scanner) or subject variability in arousal and use of strategy between sessions (64). Attention and arousal can modulate responses and influence brain activation (65), hence contributing to changes in the LI values in memory encoding. Other possible influences are differences in cognitive strategies used during the memory task to encode the words (66), or differences in performance (i.e., successful vs. unsuccessful memory), as a function of developmental change.

In contrast to memory encoding, memory retrieval LIs were not stable across runs, but showed good stability across sessions. There may not have been sufficient number of trials to capture stability of event-related LI values across runs, but the stability of retrieval LIs across sessions suggests good inter-session reliability, and is therefore promising. Stability of memory LIs should be confirmed in adults, who may show a more lateralised pattern of activation (31) and for whom ICC measurement might be appropriate. Overall, the stability of language and memory retrieval LI values across separate scanning sessions suggests reliability of these measures, and is a promising indicator of reproducibility of this paradigm.

### Implications for Future Clinical Applications

This fMRI paradigm has multiple advantages over current neuroimaging tasks. First, the combined language/memory aspect of the paradigm offers pre-operative mapping of both networks in a time-, and cost-effective manner. Memory fMRI administered in conjunction with language fMRI could provide a better guide for tailored resections, particularly in the temporal lobe, and help predict outcome. This paradigm can be used to shed light on how the two systems interact in cases of early temporal lobe-related abnormality, and explore whether lateralisation for memory and language are interdependent. Whereas co-lateralisation of language and memory functions has been demonstrated in healthy adults, whereby those with language dominance in the left hemisphere also show left lateralisation for verbal memory (67), this is less clear in children. Moreover, patients with DA exhibit severe and selective impairment in recall memory but good preservation of language skills, especially vocabulary, and other aspects of semantic memory. This indicates that the hippocampus is not crucial for the development and maintenance of language functions and semantic memory [see Elward and Vargha-Khadem (68) for a review]. The relation between language and memory networks is therefore unclear at this stage, and may critically depend on long-term auditory verbal memory (69). Although this novel paradigm investigates language and memory processes separately, it does provide an indication of the interaction between these two networks, and, potentially, of the status of functional reorganization in the context of age at onset of brain damage.

Second, the paradigm enables examination of fMRI activation related to both memory encoding and retrieval, thus providing a more robust mapping of memory-related networks, as both phases are dependent on hippocampal involvement (70, 71). Moreover, obtaining robust activation in the hippocampus at the individual level has proven challenging across fMRI studies (71, 72), but a wider approach to memory mapping involving two memory phases (encoding and retrieval) may increase the chances of capturing such an effect.

Third, this paradigm investigates activity related to recall memory, as opposed to recognition, for a more fine-grained examination of the hippocampal-neocortical network (39, 73–75). Failure to show robust activation in hippocamal regions in some fMRI studies may be due to the recognition nature of the tasks often employed, which may rely on other subregions of the MTL. Word-stem cued-recall tasks have been used by previous fMRI studies and show activation in healthy adults in several regions that are associated with successful recall, namely bilateral parietal cortex, bilateral medial temporal lobe, including the hippocampi, and left temporal cortex (45, 46, 76, 77). By contrast, adult patients with epilepsy show deficits in word-stem recall (78), making this task potentially sensitive to the identification of network abnormalities.

Fourth, the design of the paradigm permits investigation of fMRI data through both block and event-related analyses. Block analyses allow examination of brain activity related to memory effort, irrespective of performance, whereas event-related analyses examine memory success specifically, and are particularly relevant for predicting memory outcome in the clinical setting. Together, the features of this paradigm make it particularly useful for the investigation of pre-operative memory networks and for the prediction of memory outcome in TLE.

Lastly, the parallel versions of the paradigm allow systematic comparisons between performance across two time points. This paradigm can be administered before and after surgical intervention, and such clinical follow-up can provide indication of the impact of surgery on the functional organization of language and memory. Non-specific effects of test-retest can be controlled for by including a healthy controls group scanned across the same time-points as the patient group (79). A mixed ANOVA using a flexible factorial design (80) models the changes in brain activation at two time-points, whilst controlling for between-subjects and between-group variance (55). The inclusion of a control group at two time-points therefore allows adequate use of the parallel versions of the fMRI paradigm in patient groups.

Following the development of this protocol and its validation in a group of typically-developing children, confirmation of the findings is required by administering the protocol to a larger sample in order to confirm the feasibility of this tool for clinical purposes. Moreover, further work is required to validate the ability of the protocols to predict memory impairments after surgery by investigating post-surgical outcome in children with TLE. Overall, this paradigm has the potential to enhance clinical practice for pre-operative examination in TLE.

## Limitations

Despite efforts to reduce the effect of priming, it is possible that the retrieval of words is still influenced by some level of automatic retrieval, or echoic memory. Another limitation relates to the short delay between encoding and retrieval phases (50 s). The attribution to long-term memory with such delay could be disputed, but methodological considerations were put in place to insure this. The baseline task involving active and overt response prevents subvocal rehearsal and maintenance of information in short-term memory. It is possible that a longer delay between encoding and recall phases of memory is more sensitive for the investigation of hippocampal-related brain activation, but this comes with the pitfall of longer scanning time, especially with children.

The current acquisition settings were selected on the basis of a prior pilot study aimed at optimizing the acquisition sequences for pre-surgical fMRI. However, we recognize that different fMRI acquisition settings could alter brain activity measurements, and careful piloting is necessary.

Low statistical thresholds (*p* < 0.001, uncorrected) were used for memory analyses to visualize brain activation in subregions of the MTL. Whereas components above such low threshold might be labeled as noise, the findings were consistent with hypotheses postulated on the basis of prior studies in adults. In addition, small volume corrections were subsequently applied to the hippocampal region to correct for multiple comparisons. In addition, LI calculation was carried out independent of an arbitrarily defined threshold. LI values for language and memory retrieval showed good inter-session reliability, providing their promising use in single-subject level analysis which has crucial implications in the clinical context.

Despite the above limitations, the present findings provide evidence of the utility of this new paradigm for the examination of memory network in TLE. We pursued hippocampal-driven analyses based on *a priori* hypotheses, and using anatomically-constrained masking. As a result of the precautionary measures taken for the analyses, we are confident that the present findings are robust and appear promising.

## Conclusions

We present a novel fMRI paradigm to map language and verbal memory functions, as well as the interaction between them, *within one scanning session*. Other advantages of this fMRI protocol are (a) assessment of both encoding- and retrieval-related neural networks, (b) recall-based retrieval to increase hippocampal recruitment, and (c) overt responses allowing the investigation of neural networks that support successful memory specifically. This paradigm was developed to provide more precise information on neural networks subserving functions at risk, and to offer improved input to surgical decision-making in pediatric TLE. Finally, the parallel versions of the paradigm provide the means to compare language and memory activations pre- and post-surgical intervention.

## Data Availability Statement

The present script for the language/memory fMRI paradigm, as well as the scripts for pre-processing and data analyses are available upon request from the corresponding author.

## Ethics Statement

This study was ethically approved by the UCL Research Ethics Committee (project number 7447/002), and was conducted in accordance with the World Medical Association Declaration of Helsinki.

## Author Contributions

SB acquired data at time 1 of the study. FB acquired data at time 2 of the study, for test-retest measurements. All authors contributed to manuscript revision, read, and approved the submitted version.

### Conflict of Interest

The authors declare that the research was conducted in the absence of any commercial or financial relationships that could be construed as a potential conflict of interest. The handling Editor declared a shared affiliation, though no other collaboration, with one of the authors (SB).
