# Supplementary material for: A Functional MRI Paradigm Suitable for Language and Memory Mapping in Pediatric Temporal Lobe Epilepsy
Source: Front Neurol. 2020 Jan 10;10:1384. doi: 10.3389/fneur.2019.01384 (PMC6966885; doi:10.3389/fneur.2019.01384)
Supplement: Supplementary file 1 [file Data_Sheet_1.PDF]

Supplementary Material

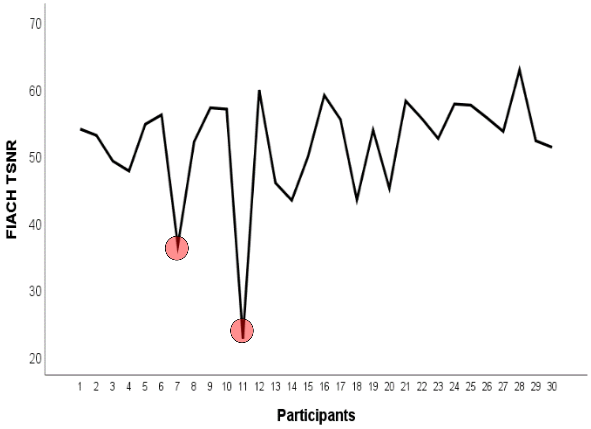

Supplementary Figure S1. FIACH tSNR (measure of deviation of realigned images) across 30 participants. The red circles identify the two participants who were excluded from the analyses due to high level of in-scanner motion.

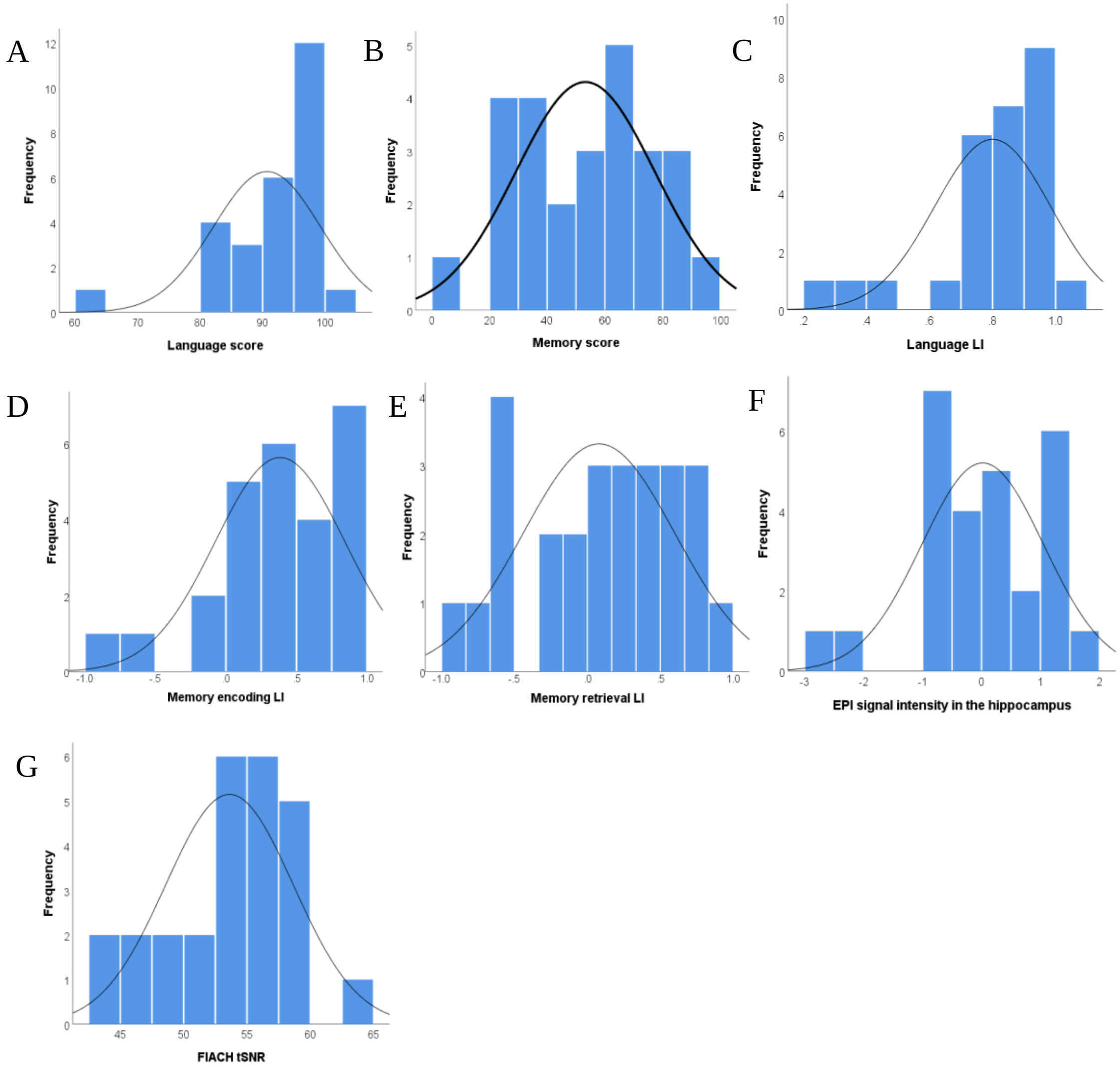

Supplementary Figure S2. Distribution plots for (A) in-scanner language performance, (B) in-scanner memory performance, (C) language LIs, (D) memory encoding LIs, (E) Memory retrieval LIs, (F) EPI mean signal intensity in the hippocampus, and (G) FIACH tSNR.

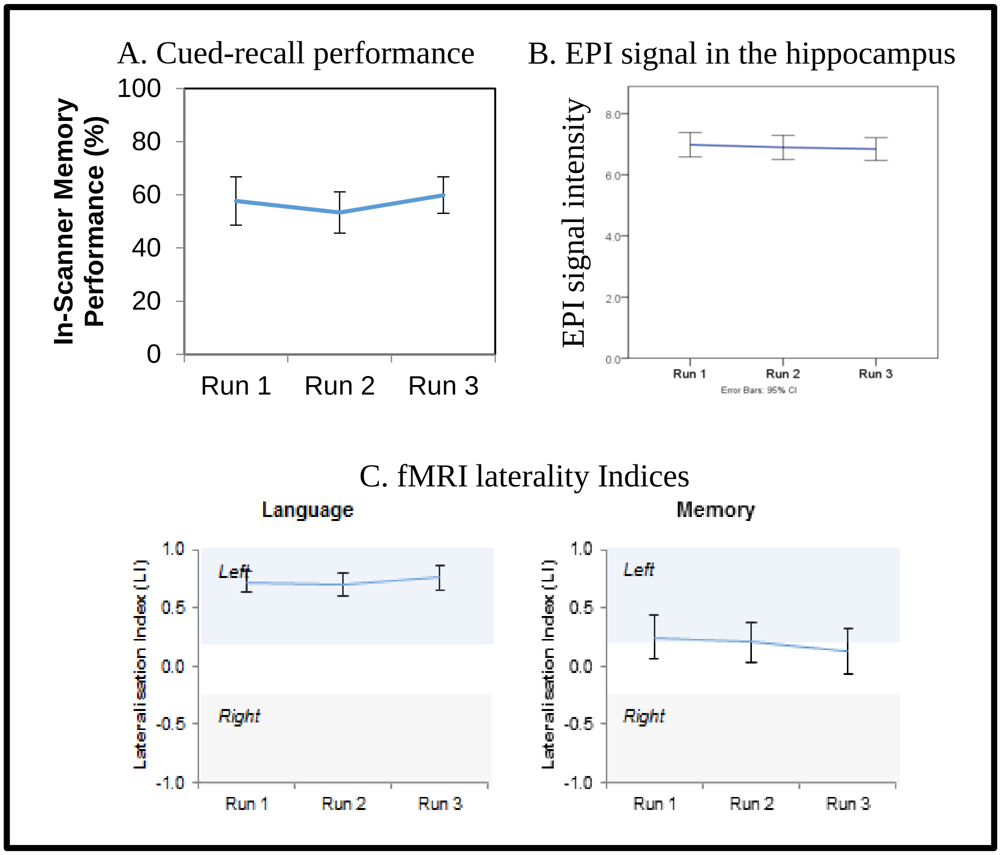

Supplementary Figure S3. Reproducibility of the paradigm. (A) Cued-recall performance across runs. (B) EPI mean signal intensity (in arbitrary units) in bilateral hippocampi across runs. (C) Group-Level Lateralisation Indices for Language (Broca's area ROI mask) and Memory (hippocampus ROI mask) in each run (errors bars are 95% CIs).

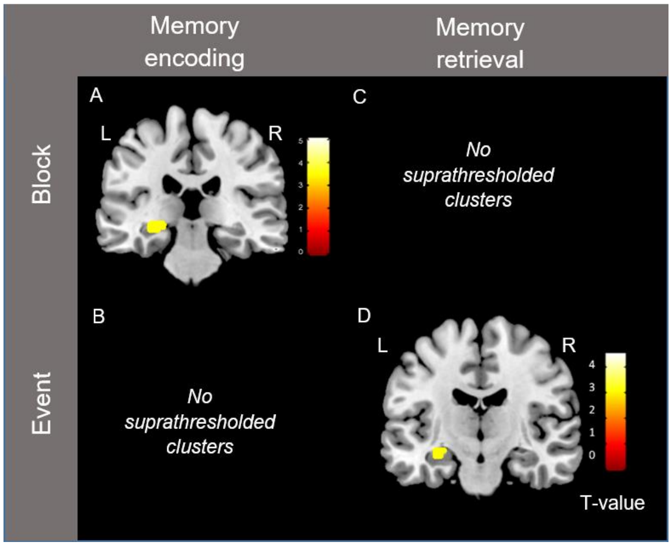

Supplementary Figure S4. Group-level activations for memory encoding and retrieval, for block and event-related analyses, shown separately. These ROI analyses are restricted to 2600 voxels within the hippocampus, and reported at a height threshold of $p<0.05$ (FWE corrected). (A) Hippocampal block-activation during memory encoding. (B) Event-related activation for subsequent memory. (C) Block-activation during memory retrieval (contrast Memory *vs*. Language). (D) Event-related activation during successful retrieval.

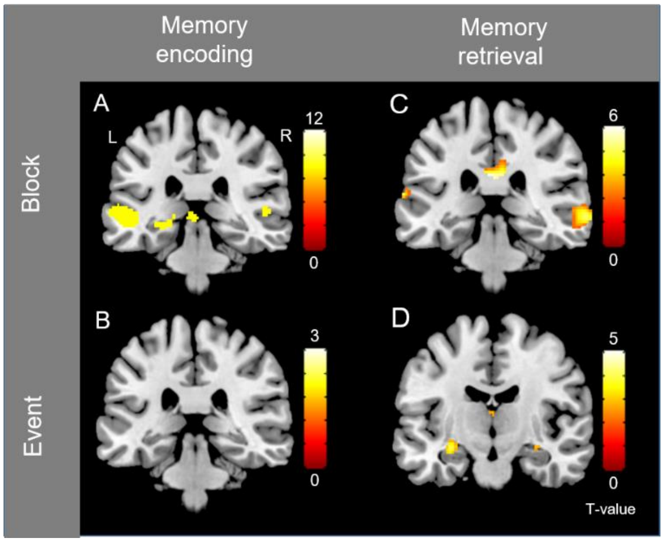

Supplementary Figure S5. Group-level activations for memory encoding and retrieval, for block and event-related analyses, shown separately, with age and gender as nuisance regressors.

Supplementary Table S1 Matched properties between the experimental and the clinical paradigm.

	Clinical		Experimental				
	M	*SD*	*M*	*SD*	*t*	df	*p* value
Word frequency	12	3	13	3	0.299	138	0.765
Concreteness	600	3	594	3	1.489	131	0.139
Familiarity	578	5	568	5	1.641	136	0.103
Imageability	606	3	600	3	1.319	135	0.189

Supplementary Table S2 Learning and memory standard scores on the CMS[1].

	M	SD
Learning	96	16
Delayed recall	99	17
Delayed recognition	99	16

Supplementary Table S3 Memory fMRI performance (% correct recall minus false alarms).

	Version A	*Version B*	*Combined versions*
Run 1	53	63	56
Run 2	49	60	53
Run 3	60	61	58
Combined runs	54	60	56

[1] Six participants were older than the standardisation norms (8 to 16 years old). To calculate standard scores for those six participants, we used the standard scores of 16 year olds.
